# Depression and Its Predictors among Diabetes Mellitus Patients Attending Treatment in Hawassa University Comprehensive Specialized Hospital, Southern Ethiopia

**DOI:** 10.1155/2020/7138513

**Published:** 2020-01-22

**Authors:** Bereket Beyene Gebre, Suzan Anand, Zebene Mekonnen Assefa

**Affiliations:** ^1^School of Nursing, College of Health Science, Hawassa University, Southern Ethiopia, Ethiopia; ^2^Department of Nursing and Midwifery, College of Health Science, Jimma University, Southwest Ethiopia, Ethiopia; ^3^College of Health Science, Department of Nursing, Wolkite University, Southern Ethiopia, Ethiopia

## Abstract

**Background:**

Patients with diabetes mellitus are at twice the risk of developing depression than the general population. The coexistence of diabetes and depression largely contributes to increased morbidity and mortality and results in high healthcare cost.

**Objective:**

The aim is to assess severity of depression and its determinants in diabetes outpatients at Hawassa University Comprehensive Specialized Referral Hospital, southern Ethiopia.

**Methods:**

An institutional-based cross-sectional study was done using a systematic sampling method. To assess the magnitude of depression, the patient health questionnaire-9 scale was used. Then, the data were entered into EpiData version 3.1 and exported to SPSS version 20 software. Binary logistic regression was used to assess the association between dependent variable and independent variables.

**Results:**

The magnitudes of depression were found to be 41.5%. The potential predictors were adhering to alcohol intake (adjusted odds ratio, AOR = 3.71, 95% CI: 1.52, 9.06), loss of someone very close or spouse (AOR = 6.83, 95% CI 3.07, 15.19), having no social support (AOR = 3.68, 95% CI: 1.63, 8.29), not adhering to the recommended dietary regimen (AOR = 6.83, 95% CI 3.07, 15.19), not adhering to physical activity (AOR = 4.1, 95% CI: 1.86,9.014), not adhering to medication (AOR = 4.2, 95% CI: 1.7, 10.31), and not having raised blood pressure of 140/90 mmHg and above (AOR = 7.42, 95% CI: 3.40, 16.17).

**Conclusion:**

Depression was a common comorbidity associated with diabetes occurring in more than four in ten of the participants.

## 1. Background

Diabetes mellitus (DM) is a group of metabolic disorders characterized by a high blood glucose level [1, 2]. Depression is a common mental health problem affecting people worldwide [3]. There is a bidirectional relationship between depression and DM suggesting that depression could be a consequence of DM and depression may also be a risk factor for DM [4].

There are various predictors of depression among DM patients such as socioeconomic, individual, behavioural and clinical related factors [5]. Literatures now show a significant association with various predictors of depression among DM patient but often goes unrecognized and untreated in developing countries [6]. In spite of the huge impact of depression in DM patient and its relevance as a public health problem, there is a gap in scientific data on the severity of depression among DM patient. The study was conducted to assess the severity of depression among patients with diabetes and can provide rich data to identify and manage patients having all individual and social predictors, most behavioral, some medication and disease related, and physical measurement predictors of depression among DM patients. The study was conducted to assess the magnitude of depression and its potential predictors among patients with diabetic mellitus, attending the DM clinic in Hawassa University Comprehensive Specialized Hospital, southern Ethiopia, 2016.

## 2. Study Area and Periods

The study was conducted in Hawassa University Comprehensive Specialized Hospital (HUCSH). It is located in Hawassa which is 285 km away from Addis Ababa. The hospital provides services in 5 case teams such as outpatient, emergency, inpatient, obstetrics and gynaecology, and operation service. There were 688 patients on follow-up having appointment during the study period. The study was conducted from March 12 to May 11, 2016.

## 3. Study Design

An institution-based cross-sectional study design using a systematic sampling method was employed. The source population was all diabetes outpatients who visited the diabetes clinic during the study period in HUCSH, southern Ethiopia. The study population comprised of selected diabetic outpatients who visited the diabetes clinic during the study period in HUCSH, southern Ethiopia. Patients aged 18 years and above were included in this study. But patients who were seriously ill were excluded from the study.

## 4. Sample Size Determination

The actual sample size for the study was determined using a single population proportion formula:

(1) $$\begin{array}{c} ni=\frac{{\left(Z_{1-\alpha /2}\right)}^{2}P\left(1-P\right)}{d^{2}} \end{array}$$

where the prevalence of depression among type 2 DM patients in 2013 in Black Lion Specialized General Hospital is 0.447 [7], *q* is the proportion of the type 2 DM population not having depression (0.553), and *d* is the marginal error (0.05); ni = ((1.96)^2^ × 0.447 × 0.553)/(0.05)^2^ = 380.

Since the sampling will be made from finite population (*N* < 10,000), it needs finite population correction. Therefore, *f* = ni/1 + ni/*N*, nf = 380/1 + 380/688 = 245, where nf is the final sample size, ni is the initial sample size determined using the formula, and *N* is 688.

By considering 10% nonresponse rate (NRR), 10%NRR = nf × 10/100 = 245 × 0.1 = 25.

So, the total sample size was calculated as follows: total sample size (Tsz) = nf + 10%of nonresponse rate, total sample size (Tsz) = 245 + 25 = 270.

## 5. Sampling Technique (Procedure)

**Figure 1:**
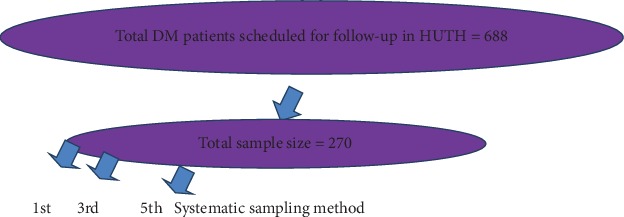
Schematic representation of sampling procedure.

In Hawassa University Comprehensive Specialized Hospital, there were 688 patients in the sampling frame which is prepared from the list of patients who had been appointed for follow-up during the study period. The sample size for the study comprised of 270 patients who were selected by systematic random sampling, calculating sampling interval *K* = (*N*/Tsz) (see Figure 1).

### 5.1. Dependent Variable

Depression in DM patients is the dependent variable.

### 5.2. Independent Variables

Age, sex, educational status, marital status, income and job status, alcohol drinking, cigarette smoking, khat chewing, physical activity practice, practices to prescribed diet regimen, health professional advice about lifestyle modifications, social problem, perceived social support, family history of psychiatric disorder, physical disability, loss of someone very close (spouse), type of DM, duration of diabetes, type of treatment, DM complication, additional illness, fasting blood glucose level (FBS), medication adherence score and practice to monitor blood glucose level, body mass index (BMI), waist circumference (WC), and blood pressure (BP) are the independent variables.

### 5.3. Operational Definitions

No depression is a self-reported depressive symptom identified by the PHQ-9 scale with a score < 5.

Depression is a self-reported depressive symptom identified by the PHQ-9 scale with a score ≥ 5.

### 5.4. Data Collection, Processing, and Analysis

#### 5.4.1. Data Collection Instrument

The initial English version of the questionnaire was translated into Amharic. Then, it was translated back into English independently by language experts to maintain the equivalence of the test questionnaire in Amharic. The questionnaires have six parts which are the sociodemographic information, patient health questionaire-9 scale, behavioral predictors, individual and social predictors, and disease- and medication-related predictors. The behavioral and physical measurements of predictor variables of depression among DM patients were adapted from the WHO 2014 stepwise approach to the NCD guide [8]. The valid and reliable H SCALE was also used to assess the behavioral predictors of depression [9]. The disease- and medication-related predictors of depression had been documented from the patients' medical cards. The Morisky Medication Adherence Scale having 8 valid and reliable items (MMAS-8) was used to assess patients' adherence to prescribed medication [10]. To measure physical measurement, the individual patient actual measurements by data collectors were done using a standard measuring instrument.

#### 5.4.2. Data Collection Method (Technique)

Quantitative data were collected by using a face-to-face interview and a medical record review from March 12 to April 11, 2016. Information on the sociodemographic characteristics, PHQ-9, behavioral predictors, and individual and social predictors was gathered from patients' responses to the structured questionnaire. The disease- and medication-related predictors were obtained from the medical records of the participants and verbal responses to the MMAS-8. The physical measurement predictors were assessed using the measuring instruments. The data were collected by 6 diploma nurses and 2 BSc health professional supervisors working in the hospital at the exit interview.

#### 5.4.3. Data Quality Assurance

To assure quality of the data, properly designed data collection tools were prepared and the questionnaire was pretested on 5% of the sample size in “Adare” General Hospital to check for understandability and applicability of the instrument. The pretest showed that Cronbach's *α* for the PHQ-9 scale was 0.76, H SCALE was 0.77, and Morisky Medication Adherence Score (MMAS-8) was 0.79, indicating acceptable consistency of this psychometric scale. In addition to this, two-day training was given to data collectors and supervisor. Besides, the collected data were reviewed and checked for their completeness by the supervisor and principal investigator at the end of each day.

#### 5.4.4. Data Processing and Analysis

After checking collected data visually for completeness, the responses were cleaned, edited, coded, and entered into the computer using EpiData 3.1 version. The data were then exported to SPSS version 20.0. The data were checked for missed value before analysis. The descriptive analysis including frequency and cross tabs was used to assess frequency of variables with independent variables. Binary logistic regression was carried out to assess the association of dependent variable with independent variables and to determine predictors of depression using odds ratios with 95% confidence interval. Finally, a forward stepwise logistic regression model with all independent variables having *p* value < 0.25 was fitted, and an adjusted odds ratio was calculated to identify the independent predictors of depression among DM patient.

### 5.5. Ethical Consideration

In order to follow the ethical and legal standards of scientific investigation, the study was conducted after approval of the proposal by the ethical and review board of Jimma University College of Health Science. Permission for conduct of study was obtained from HUCSH. Written informed consent was obtained from participant by assuring privacy and confidentiality. Besides, patients identified with depression have been linked to a psychiatric unit within the hospital. An individual who was unwilling to participate from the beginning or at any part of the interview was allowed to withdraw. Those participants who were diagnosed as having depression were linked to a psychiatric unit within the hospital.

## 6. Results

### 6.1. Sociodemographic Characteristics

Among the sampled 270 diabetic outpatients, 260 of them participated in the study yielding a response rate of 96.3%. Ten participants declined citing lack of time. From those interviewed, 138 (53.1%) were males. The mean age and standard deviation (SD) were 43.8 years (SD = 11.38). Those participants aged between 18-34 and 35-44 were 66 (25.4%) and 65 (25.0%), respectively (see Table 1).

### 6.2. Behavioral Factors

From the total participants, 250 (96.2%) and 228 (87.7%) never smoked cigarette and chewed khat, respectively, and 77.3% did not consume alcohol (see Table 1).

### 6.3. Individual and Social Factors

There was no family history of psychiatric illness among 234 (90%) of the respondents, and 255 (98.1%) of them did not give family history of physical disability. Besides, 88.8% reported being advised by health professionals on lifestyle modification (see Table 1).

### 6.4. Disease- and Medication-Related Factors

The majority of patients suffered from type 2 DM 191 (73.5%). Responses to questions about monitoring of their FBS revealed that 222 (85.4%) did not have even weekly monitoring of FBS. From the total participants, 183 (70.4%) were adherent to prescribed medication (see Table 2).

### 6.5. Physical Measurement Factors

The blood pressure recordings of the participants showed that 136 (52.3%) had blood pressure < 140/90 mmHg, and 134 (51.5%) had BMI 25-29.99 kg/cm^2^ (see Table 2).

### 6.6. The Severity and Level of Depression among DM Patients

#### 6.6.1. Magnitude of Depression

**Figure 2:**
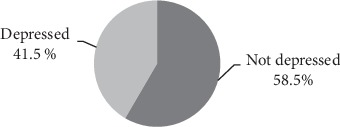
Magnitude of depression among DM patient in HUCSH, southern Ethiopia, 2016.

From the 260 study participants, 108 (41.5%) patients were depressed with the PHQ-9 scoring <5 and 152 (58.5%) did not show symptoms of depression with PHQ‐9 ≥ 5 (see Figure 2).

#### 6.6.2. Level of Depression

**Figure 3:**
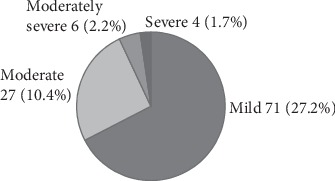
Level of depression among DM patients in HUCSH, southern Ethiopia, 2016 (*n* = 260).

Out of the 108 (41.5%) respondents who were categorized as depressed, 71 (27.2%) were mildly depressed (see Figure 3).

### 6.7. Individual and Social Factors as Predictors of Depression

From sociodemographic variables in the binary logistic regression, sex, age, educational status, and income had significant association with depression among DM patients. From the behavioral variables, use of alcohol, physical activity, and dietary practice were significant associated. Among individual and social variables, social problem, social support, and loss of someone close (spouse) were found to be associated significantly (see Table 1).

### 6.8. Clinical Factors as Predictors of Depression

Results of disease- and medication-related factors in the binary logistic regression showed that the FBS level, type of treatment, comorbid diseases, DM complication, and medication adherence were found to be significantly associated with depression.

This study also showed that from the physical measurements, those who had BP 140/90 mmHg and BMI 25-29.99 kg/cm^2^ were found to be depressed (see Table 2).

#### 6.8.1. Potential Predictors of Depression in DM

From 28 independent variables, 23 variables with *p* < 0.25 were entered into forward stepwise binary logistic regression. Model fitness is also tested by the Hosmer-Lemeshow test having a value of 0.844 indicating that the model was fitted. Finally, 7 variables predict independently. These are consumption of alcohol, loss of close relative/spouse, lack of social support, not adhering to diet, poor physical activity, nonadherence to prescribed medication, and raised blood pressure.

## 7. Discussion

In this study from the 260 patients who participated, the findings revealed that the magnitude of depression among DM outpatient in HUCSH was found to be 41.5%. However, the magnitude of depression among DM varies and ranges from 2 to 28% in USA [11], 34% to 39% in Croatian, 19% to 39% in English [12], and 34.8% in Uganda [13]. It is low compared to the finding of this study. This may be due to variation in the developments of the country with better healthcare. This study is in line with the study done in Sudan with a magnitude of 44% [14], 43.6% in JUTH, southwest Ethiopia [15]. But it is slightly higher than the study done in Saudi Arabia having a magnitude of 49.6% in 2013 [16]. This variation in the magnitude may be due to sociopolitical situation and variation in measurement scales of depression especially in the study reported in Saudi Arabia. The degree and magnitude of depression among DM in the current study result is almost consistent with the study done in 2013 in Black Lion Teaching Hospital, Ethiopia [7]. So using a psychometric tool to assess the presence of depression should be initiated for patients having a follow-up in a diabetic clinic.

In this study among those participants who reported poor physical activity, 60.7% were depressed compared to those who did not practice physical exercises (AOR = 4.1). This finding is higher than the study done in Qatar in 2010 (AOR = 1.85) [17]. This may be due to variation in measurement scale of physical activity practice, study design, time frame, and socioeconomic level of the participants. In this study, measurement scale used seems difficult to measure physical activity than the measurement used in the Qatar study in which it seems simple and most of the participants may adhere to this measurement scale of physical activity.

Regarding recommended dietary practice regimen, among individuals who were not adhered to dietary modifications, 62.2% were depressed. It is in line with the study done in South Korea in 2013 indicating that 2/3rd (66%) of patients who did not follow recommended diet changes were depressed [18]. This supports the assumption that unregulated dietary habits in diabetes have an impact on low mood. The findings in this study may also be due to lower education level of the participants.

Consumption of alcohol was found in 62.7% who were depressed (AOR = 3.7). However, the study done in Korea from 2005 to 2012 showed that current alcohol consumption did not show any statistically significant association with depression among DM patients [19]. But this is contrary to the report from Delhi, India, in 2013, in which alcohol consumption was a predictor for depression in DM patients [20]. But another study in 2012, from India, revealed depression was present in 58% individuals with substance abuse [21]. This study finding is high compared to the Indian study. This may be because the Indian study was a community-based study, with small sample size and geriatric depression scale (GDS) to diagnose depression. Alcohol consumption remains controversial as many scientific studies suggest that alcohol had a high risk than benefit. Some of the risks due to alcohol consumption are glucose intolerance, acute pancreatitis, induces fasting hypoglycaemia, impairs the ability to recognize, precipitates diabetic ketoacidosis, have a synergetic effect for complication of DM, leads to weight gain, elevated glucose level, and impairs adherence to DM self-care behavior. This implied that provision of advice from a healthcare provider to the patient to minimize intake of alcohol was encouraged.

In our study among those with poor social support, 51.7% had depression (AOR = 3.68). But the study done in 2014 in Morocco indicated that from those who had no social support (security), 43.3% had depression [22]. The higher result in this study may be due to low social support for DM patient in the community, absence of social support in the hospital, and low educational status of the participants to be aware about the disease and its process. It may also be due to the small sample size with different measurement scales in the Moroccan study. So, strengthening social support has to be encouraged.

From the participants who had lost very close relative/spouse, 65.2% of them were depressed (AOR = 6.83). But this study finding is higher than the study done in Malaysia in 2012 which indicated that from those who had lost spouse, 31.8% had depression (AOR = 4.62) [23]. This may be in Malaysia PHQ scale ≥ 10 was used to assess depression. Besides, this study result is also higher than the study done in 2013-14 in Uganda (AOR = 2.36). Our study finding is also high compared to the study done in Uganda using a Mini-International Neuropsychiatric Inventory (MINI) measurement scale [13]. The low educational background of the participants may contribute to poor coping skills for this situation.

The data analyzed in the study showed that from those who had poor adherence to prescribed medication, 71.4% had depression. The study done in Palestine in 2012 showed that from among those with poor medication adherence, 51.5% of them had depression [24]. The higher result in this study may be due to low educational status of the participants that contribute to lack of awareness and neglect of professional advice from healthcare providers about compliance to medication, or it may be due to inadequate advice from health professionals about medication adherence, lack of support from hospital staff on medication compliance, for patients attending DM clinics. Variations in findings may also be due to different measurement scales used to assess depression. So, encouraging patients to adhere for prescribed medication should be strengthened.

Our study results showed that from those who had raised BP ≥ 140/90 mmHg, 66% were depressed (AOR = 7.42). A study from Pakistan in 2010 using HADS showed that in an individual who had raised BP ≥ 140/90 mmHg, the probability of an individual to be depressed will increase by 2.31 as compared to those having BP < 140/90 mmHg [21]. Our finding shows that association of depression with BP is higher than that of the Pakistan study. This may be due to the fact that behavioral predictors in Pakistan were not assessed in the Pakistan study. But in our study, it accounts for a large share for an individual to be depressed. So, it may contribute to an individual being more likely to be depressed. It may also be because patients with diabetes-related complications are referred from surrounding health institutions to HUCSH.

The limitation in this study is the recall bias for the questions like the PHQ scale, subjective and social desirability bias for questions about alcohol intake, physical activity, dietary regimen, and medication.

## 8. Conclusion

Depression is a common condition associated with diabetes mellitus. In this study, the magnitude of depression was high with the highest percentage of participants having mild-type depression.

Potential predictors of depression among diabetes were consumption of alcohol, failure to practice recommended physical activity, not practicing a recommended dietary regimen, loss of very close relative/spouse, poor social support, nonadherence to prescribed medication, and having raised blood pressure ≥ 140/90 mmHg. So, patients should be assessed and managed as early as when patients arrive for treatment follow-up in the clinic for a good clinical outcome.

## Figures and Tables

**Table 1:** Binary logistic regression model for individual and social factors as predictors of depression among DM patient in HUCSH, southern Ethiopia, 2016 (*n* = 260).

		Depression		COR, 95% CI
		No, *n* (%)	Yes, *n* (%)	
1	*Sociodemographic factors*			
	Sex			
	Male	95 (68.8%)	43 (31.2%)	**1**
	Female	57 (46.7%)	65 (53.3%)	**2.5** (1.5, 4.18)
	Age in years			
	18-34	52 (78.8%)	14 (21.2%)	**1**
	35-44	47 (72.3%)	18 (27.7%)	1.4 (0.63, 3.17)
	45-54	35 (51.5%)	33 (48.5%)	3.5 (1.64, 7.47)
	55 and above	18 (29.5%)	43 (70.5%)	8.8 (3.96, 19.8)
	Marital status			
	Single	23 (67.6%)	11 (32.4%)	**1**
	Married	120 (56.6%)	92 (43.4%)	1.6 (0.74, 3.45)
	Widow/divorced	9 (64.3%)	5 (35.7%)	1.16 (0.31, 4.2)
	Educational status			
	Illiterate	35 (44.3%)	44 (55.7%)	2.79 (1.58, 4.9)
	Read & write only	17 (47.2%)	19 (52.8%)	2.48 (1.18, 5.2)
	Formal education	100 (69.0%)	45 (31.0%)	**1**
	Job status			
	Governmental	52 (65%)	28 (35%)	**1**
	Private employ	20 (64.5%)	11 (35.5%)	1.02 (0.42,2.4)
	Merchant	23 (59%)	16 (41%)	1.29 (0.58, 2.8)
	Student	15 (83.3%)	3 (16.7%)	0.37 (0.99, 1.3)
	Housewife	25 (50%)	25 (50%)	1.85 (0.9, 3.8)
	Unemployed	6 (35.3%)	11 (64.7%)	3.4 (1.1, 10.1)
	Farmer	11 (44.0%)	14 (56.0%)	2.36 (0.94, 5.8)
	Income in birr			
	Below 13 USD	20 (36.4%)	35 (63.6%)	3.93 (1.83, 8.4)
	13-26.9 USD	43 (56.6%)	33 (43.4%)	1.72 (0.86, 3.4)
	27-38 USD	44 (68.8%)	20 (31.2%)	1.02 (0.48, 2.1)
	Above 38 USD	45 (69.2%)	20 (30.8%)	**1**
2	*Behavioral predictors*			
	Alcohol			
	Not adhered	130 (64.7%)	71 (35.3%)	**1**
	Adhered	22 (37.3%)	37 (62.7%)	3.07 (1.68, 5.6)
	Khat chewing			
	Yes	18 (56.2%)	14 (43.8%)	1.10 (0.52, 2.3)
	No	134 (58.8%)	94 (41.2%)	**1**
	Physical activity			
	Not adhered	53 (39.3)	82 (60.7%)	6.15 (3.5, 10)
	Adhered	99 (79.2%)	26 (20.8%)	**1**
	Diet regimen			
	Not adhered	59 (37.8%)	97 (62.2%)	5.89 (3.3, 10.2)
	Adhered	93 (89.4%)	11 (10.6%)	**1**
3	*Individual and social predictors*			
	Health advice			
	Yes	131 (56.7%)	100 (43.3%)	**1**
	No	21 (72.4%)	8 (27.6%)	2.0 (0.85, 4.7)
	Social problems			
	Yes	47 (48.0%)	51 (52.0%)	1.99 (1.19, 3.3)
	No	105 (64.8%)	57 (35.2%)	**1**
	Social support			
	Yes	95 (66.9%)	47 (33.1%)	**1**
	No	57 (48.3%)	61 (51.7%)	2.16 (1.3, 3.57)
	Family history of psychiatric illness			
	Yes	13 (50%)	13 (50%)	1.46 (0.65, 3.2)
	No	139 (59.4%)	95 (40.6%)	**1**
	Loss of someone very close (spouse)			
	Yes	39 (34.8%)	73 (65.2%)	6.04 (3.5, 10.4)
	No	113 (76.4%)	35 (23.6%)	**1**

**Table 2:** Binary logistic regression model for clinical factors as predictors of depression among DM patient in HUCSH, southern Ethiopia, 2016 (*n* = 260).

		Depression		COR, 95% CI
		*N* (%)	*N* (%)	
1	*Disease- and medication-related predictors*			
	DM type			
	Type I	47 (68.1%)	22 (31.9%)	**1**
	Type II	105 (55.0%)	86 (45.0%)	1.75 (0.97, 3.12)
	FBS			
	FBS: ≤126 mg/dl	105 (65.6%)	55 (34.4%)	**1**
	FBS: ≥127 mg/dl	47 (47%)	53 (53%)	2.15 (1.29, 3.58)
	Practice to monitor FBS			
	Yes (1-4 days/week)	27 (71.1%)	11 (28.9%)	**1**
	No (0 day/week)	125 (56.3%)	97 (43.7%)	1.9 (0.9, 4.03)
	Type of treatment			
	Insulin	47 (69.1%)	21 (30.9%)	**1**
	Oral medication	96 (60.8%)	62 (39.2%)	1.44 (0.78, 2.64)
	Both	9 (23.7)	29 (76.3%)	6.2 (2.47, 15.59)
	DM duration			
	5 years and below	77 (56.2%)	60 (43.8%)	**1**
	Above 5 years	75 (61%)	48 (39%)	0.82 (0.5, 1.34)
	Comorbidity			
	1 ≤ 1	67 (72.8%)	25 (27.2%)	**1**
	2 ≥ 2	85 (50.6%)	83 (49.4%)	2.61 (1.51, 4.53)
	DM complication			
	Yes	47 (40.2%)	70 (59.8%)	4.1 (2.43, 6.94)
	No	105 (73.4%)	38 (26.6%)	**1**
	Medication			
	Not adhered	22 (28.6%)	55 (71.4%)	6.13 (3.4, 11.04)
	Adhered	130 (71%)	53 (29%)	**1**
2	*Physical measurement*			
	BP ≥ 140/90 mmHg			
	Yes	42 (33.9%)	82 (66.1%)	8.26 (4.68, 14.55)
	No	110 (80.9%)	26 (19.1%)	**1**
	BMI in kg/cm^2^			
	Below 18.5	12 (57.1%)	9 (42.9%)	0.6 (0.22,1.66)
	18.5-24.99	32 (65.3%)	17 (34.7%)	0.42 (0.19,0.94)
	25-29.99	83 (61.9%)	51 (38.1%)	0.49 (0.26,0.93)
	30 and above	25 (44.6%)	31 (55.4%)	**1**
	Waist circumference			
	≤94 cm for male	63 (75.9%)	20 (24.1%)	0.13 (0.06,0.28)
	≤80 cm for female	38 (60.3%)	25 (39.7%)	0.28 (0.13,0.59)
	>94 cm for male	33 (61.1%)	21 (38.9%)	0.27 (0.12,0.59)
	>80 cm for female	18 (30%)	42 (70%)	**1**

NB. 1: reference category.

## Data Availability

The data supporting the finding was attached to public repositories.

## Abbreviations

BDI: Beck depression inventory scale
BMI: Body mass index
BP: Blood pressure
CI: Confidence interval
DBP: Diastolic blood pressure
DM: Diabetes mellitus
DSM: *Diagnostic Statistical Manual of Mental Disorders*
EDID: European Depression in Diabetes
et al: Et alia
FDRE: Federal Democratic Republic of Ethiopia
HPN: Hypertension
kg/cm^2^: Kilogram per centimetre square
JUTH: Jimma University Teaching Hospital
HUCSH: Hawassa University Comprehensive and Specialized Hospital
MOH: Ministry of Health
NGOs: Nongovernmental organizations
PHQ: Patient health questionnaire.
